# Patient Experiences of Decentralized HIV Treatment and Care in Plateau State, North Central Nigeria: A Qualitative Study

**DOI:** 10.1155/2017/2838059

**Published:** 2017-02-26

**Authors:** Grace O. Kolawole, Hannah N. Gilbert, Nancin Y. Dadem, Becky L. Genberg, Patricia A. Agaba, Prosper Okonkwo, Oche O. Agbaji, Norma C. Ware

**Affiliations:** ^1^Infectious Diseases Unit, Jos University Teaching Hospital, Jos, Nigeria; ^2^Department of Global Health and Social Medicine, Harvard Medical School, Boston, MA, USA; ^3^Department of Health Services, Policy & Practice, School of Public Health, Brown University, Providence, RI, USA; ^4^Department of Family Medicine, Jos University Teaching Hospital, University of Jos, Jos, Nigeria; ^5^AIDS Prevention in Nigeria, Abuja, Nigeria; ^6^Department of Medicine, Jos University Teaching Hospital, University of Jos, Jos, Nigeria; ^7^Department of Medicine, Division of Global Health Equity, Brigham and Women's Hospital, Boston, MA, USA

## Abstract

*Background.* Decentralization of care and treatment for HIV infection in Africa makes services available in local health facilities. Decentralization has been associated with improved retention and comparable or superior treatment outcomes, but patient experiences are not well understood.* Methods. *We conducted a qualitative study of patient experiences in decentralized HIV care in Plateau State, north central Nigeria. Five decentralized care sites in the Plateau State Decentralization Initiative were purposefully selected. Ninety-three patients and 16 providers at these sites participated in individual interviews and focus groups. Data collection activities were audio-recorded and transcribed. Transcripts were inductively content analyzed to derive descriptive categories representing patient experiences of decentralized care.* Results. *Patient participants in this study experienced the transition to decentralized care as a series of “trade-offs.” Advantages cited included saving time and money on travel to clinic visits, avoiding dangers on the road, and the “family-like atmosphere” found in some decentralized clinics. Disadvantages were loss of access to ancillary services, reduced opportunities for interaction with providers, and increased risk of disclosure. Participants preferred decentralized services overall.* Conclusion. *Difficulty and cost of travel remain a fundamental barrier to accessing HIV care outside urban centers, suggesting increased availability of community-based services will be enthusiastically received.

## 1. Introduction

Immediate treatment of all persons found to be HIV-infected—the “test-and-treat” approach to HIV service delivery [1]—requires rethinking traditional, clinic-based models of HIV care, particularly in resource-scarce settings. A framework to guide this rethinking may be found in the concept of “differentiated care” [2].

The differentiated care framework is a response to both the growing number of individuals accessing antiretroviral therapy as the test-and-treat strategy takes hold, and a recognition that more and more individuals taking ART are clinically stable and virally suppressed. Not every patient requires frequent follow-up and laboratory monitoring, suggesting that services may be “differentiated” to meet patients' varying needs and preferences.

To simplify care experiences for patients, services based on differentiated care models are increasingly located in communities, rather than large HIV-specialty clinics. New community-based services, such as home and mobile van HIV testing and counseling, point-of-care CD4 and viral load testing, ART refill groups, and community drug distribution points (CDDPs) [3–12], have their roots in the movement to decentralize ART initiation and follow-up care.

Decentralization was introduced by the World Health Organization in 2004 as a strategy for expanding delivery of ART from tertiary to secondary and primary health care settings, closer to patients' homes [13, 14]. Since then, research on decentralized HIV treatment and care in Africa has documented expanded service access, improved retention, and comparable or superior treatment outcomes [15–24]. However, patient experiences of decentralized care are not well understood. A better understanding of patient experiences can help to improve decentralization efforts and inform new models of differentiated care. Using data from a qualitative research study, this paper asks the question: How do patients receiving HIV care and treatment as part of the Plateau State Decentralization Initiative, north central Nigeria, describe their experiences of services?

## 2. Methods

### 2.1. Research Setting

With more than 160 million persons, Nigeria is the most populous country in Africa. HIV prevalence in Nigeria has declined from a high of 5.8% in 2001 to 3.1% in 2014 [25]. Despite a relatively low current prevalence rate, Nigeria's large population means its HIV burden is the second highest world-wide, with approximately 3.2 million individuals living with HIV [26, 27]. HIV prevalence in Nigeria varies substantially by region. In Plateau State, site of this research, the rate of HIV prevalence was just under 8% in 2010 [26].

At the time of this study, north central Nigeria was caught up in the civil unrest that has continued to plague the northeastern region of the country. Since 2003, the Islamist militant group Boko Haram has been violently seeking to establish an Islamic state and Sharia law in the country [28]. For a time, the campaign of violence reached beyond Nigeria's northeastern region, into Plateau State, home of the Plateau State Decentralization Initiative.

### 2.2. The Plateau State Decentralization Initiative

**Figure 1:**
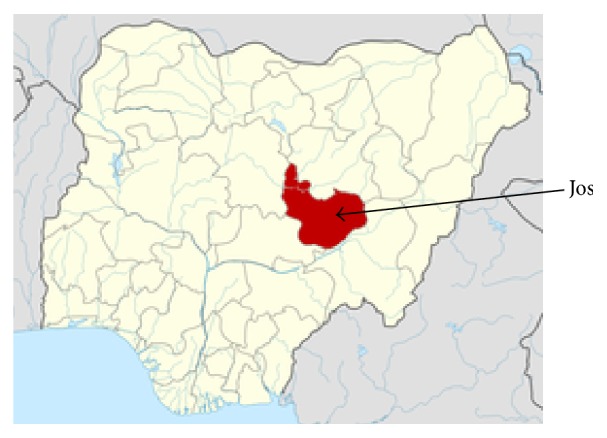
Map of Nigeria showing Plateau State (in red) and the City of Jos. https://upload.wikimedia.org/wikipedia/commons/thumb/6/66/Nigeria_Plateau_State_map.png/250px-Nigeria_Plateau_State_map.png.

As part of its national ART scale-up effort, Nigeria launched a full Decentralization Initiative in north central Plateau State in 2007. The initiative grew out of the HIV treatment program at Jos University Teaching Hospital (JUTH), in the state's capital city of Jos (see Figure 1). This large city serves as the “hub” for HIV care and treatment for the north central geopolitical zone of Nigeria.

**Figure 2:**
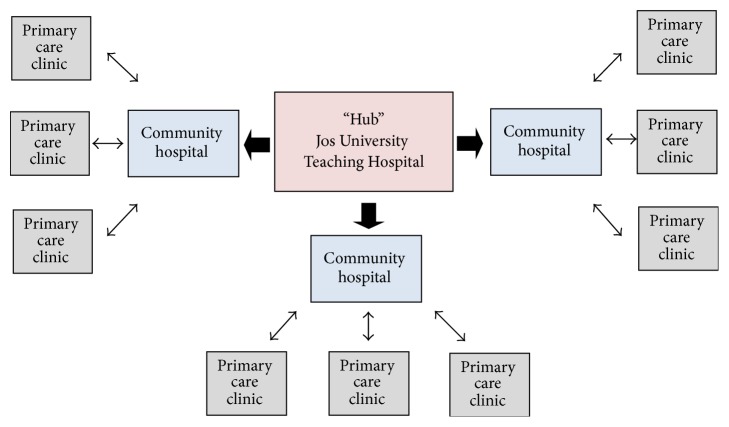
The Plateau State Decentralization Initiative's “hub-and-spoke” model of decentralized care.

The HIV treatment program at JUTH is a major public resource for HIV services in the area, providing comprehensive HIV treatment and care for the city of Jos and serving as a referral center for Plateau and neighboring states. The Decentralization Initiative's “hub-and-spoke” model of care aimed to replicate the JUTH program at smaller community hospitals in the surrounding region and to upgrade services for HIV available through the primary care facilities attached to each of these hospitals (see Figure 2). Community hospital clinics would offer clinical staging, ART initiation, and clinical follow-up, as well as HIV testing. Primary care facilities would carry out prevention education activities and HIV testing, referring individuals with positive test results to the decentralized hospital clinics for further testing and follow-up.

The initiative was implemented across Plateau State's northern, central, and southern zones over five years. When complete, the resulting “hub-and-spoke” network consisted of 47 primary care facilities and 13 community hospital clinics (the “spokes”), arranged around the HIV-specialty clinic at JUTH (the “hub”). Public and private (including faith-based) affiliations are represented in the group of community hospital clinics, which serve from fewer than 100 to more than 1500 patients each. As decentralized services became available, patients being seen at the JUTH clinic were given the option of transferring their care to a facility nearer their homes.

### 2.3. Study Design and Sampling

This qualitative study examined patient experiences of decentralized HIV treatment and care in the Plateau State Decentralization Initiative. A purposeful, facility-level sampling strategy was used to select for study participation five of the 13 decentralized clinics in the hub-and-spoke care network [29]. Clinic sites were selected to represent the types of clinics included in the larger network. One site was a public, government clinic; one was a private clinic; and three were faith-based. The sites were also selected to be geographically diverse, with three located in the northern, one in the central, and one in the southern zone of Plateau State.

### 2.4. Study Participants

Ninety-three (*N* = 93) HIV-infected individuals receiving HIV treatment and care at one of the five decentralized clinics included in the study sample (“patient participants”) and 16 health care professionals providing HIV care and treatment at these same clinics (“provider participants”) took part in the study. All patient participants had chosen to transfer their HIV services from the JUTH hub to a decentralized site as part of the Decentralization Initiative. Participation in the study was voluntary; all patient participants were individuals who volunteered to take part after hearing descriptions of the study and invitations to participate extended by health care providers and/or administrative staff. All patients who were identified were subsequently invited and agreed to participate.

Provider participants were all individuals who had experienced the transition to decentralized HIV care and treatment while working at the local clinic. Provider participants meeting this criterion were referred to the researchers by clinic administrative staff. The researchers described the study to referred individuals and extended an invitation to participate. All providers invited agreed to take part in the study.

Consent to participate in the study was confirmed orally for each participant at the beginning of each data collection session, before data collection began.

### 2.5. Data Collection

Data collection consisted of individual, open-ended interviews and focus group discussions (FGDs). Seventy patients and all 16 providers took part in interviews. Twenty-three patients took part in four focus group discussions, with 5-6 patients in each. Data were collected by the primary and third authors (GK and ND).

Individual interviews followed an interview guide organized by topics. The same set of topics was covered in each interview, but the questions corresponding to each topic were tailored to fit the situations of individual respondents. Patient interviews covered (a) reasons for transferring care to a decentralized site, (b) experiences of decentralized care, (c) preference for type of care site (decentralized or “hub”), and (d) comparisons of “hub” versus decentralized care. Provider interviews elicited (a) descriptions of decentralized treatment and care activities, (b) assessments of the strengths and weaknesses of care provided, (c) perceptions of patient responses to care provided, and (d) perceived successes and shortcomings of the Decentralization Initiative. Interviews were carried out English or the local language (Hausa), in private spaces at the decentralized care sites. They lasted an average of 45 minutes; all were audio-recorded with permission.

The purpose of the focus groups was to elicit group-level information and greater insight on points emerging repeatedly from the individual interviews, so as to gauge the extent to which these represented more widely shared, or “thematic” views on the part of patients receiving decentralized care. Thus the topics covered in these discussions reflected the content of the individual interviews.

Focus groups took place in private spaces at the different decentralized care sites in the study sample and lasted an average of 45 minutes. Discussions were in English or Hausa, depending on the language preference of the participants. Each session was audio-recorded with permission from study participants.

Transcriptions of the individual interviews and focus groups were produced verbatim, directly into English, from audio files by the primary and third authors (GK and ND).

### 2.6. Data Analysis

Data from individual interviews and focus groups were analyzed inductively using a content analytic approach [30]. The goal of the analysis was to derive a set of descriptive categories representing thematic patient experiences of decentralized care.

Analysis began with review and weekly discussion of each interview transcript by authors GK, ND, and NW. The reviews identified sections of transcribed text judged to be relevant to patient experiences of decentralized care. Similar sections of text were grouped together and labeled to form coding categories. Coding categories were formalized as a codebook, consisting of 22 codes. A trained research assistant used the codebook to code the entire data set. ATLAS.ti qualitative data management software was used to support the coding process.

Coded data were examined collaboratively by authors GK, HG, ND, and NW in regular phone discussions to identify repeated content or themes. These authors then elaborated the themes into categories consisting of a label, description, and illustrations from the data. Seven categories representing patient experiences of decentralized care emerged from this analysis. These categories form the core of the study results and are presented in Results, below (A)–(G). The categories are grouped under two broad headings: (I) perceived advantages and (II) perceived disadvantages of decentralized care, compared to central, “hub” experiences. Each patient participant taking part in an individual interview was also asked to express a preference for decentralized or “hub” care.

### 2.7. Ethical Statement

This study was approved by the Committee on Human Studies at Harvard Medical School, Boston, MA, and the Jos University Teaching Hospital (JUTH) ethical clearance committee, Jos, Nigeria.

## 3. Results

### 3.1. Demographic Characteristics of Study Participants

More than three-quarters of the patient participants were women. Approximately two-thirds were married. Median age of the patient sample was 38 (IQR = 30–45). Years of education ranged from 0 to 20 years, with a median of 9 years (IQR = 6–14). A range of professional roles was represented in the provider participant group, including medical officer, nurse, counselor, pharmacist, data manager, and purchasing officer (see Table 1).

### 3.2. Overview of Study Results

Study participants pointed to advantages and disadvantages, compared to the hub, in describing their experiences of decentralized care. Advantages included saving time and money on travel to clinic visits and avoiding exposure to dangers on the road. Another reported advantage was the “family-like atmosphere” perceived in some decentralized clinics, where fewer patients and particularly caring providers set the stage for a more personalized care experience. Disadvantages cited by participants centered on lack of access to ancillary services they had at times enjoyed as hub patients, reduced opportunities for interaction with health care professionals, and, for some, increased risk of disclosure of HIV infection. Despite these disadvantages, when asked specifically, participants expressed a clear preference for decentralized services overall. These advantages, disadvantages, and preferences are described in detail in Sections 3.2.1–3.2.3 below.

#### 3.2.1. Perceived Advantages of Decentralization

*(A) Saving Money*. Transferring care to a decentralized clinic allowed participants to drastically reduce, or even eliminate, their transportation expenditures. Interviewees noted that, in addition to transport fares, the standard tab for a “hub” clinic visit encompassed paying for one or more meals while away from home and, in some cases, the additional cost of overnight accommodations. Expenses were compounded for families with more than one person attending the HIV clinic. One man observed:

…The problem of going to [the hub] is the transport fare. Because for each person, you pay 800 naira [approximately 2.50 USD]. Because it is I and my wife, they take us for 1,600 going and 1600 coming back. It is too much and that is why we came here…. (Patient Participant, Individual Interview, Male)

Interviewees offered important details about how recuperating money previously spent on clinic travel contributed to their financial and social well-being. Transferring to decentralized clinics meant people had more money available to purchase high quality foods, which supported their overall health. They were also able to apply the money saved on transport fees to important familial and social obligations. In the words of one:

…Because [the clinic] is closer [to home], you can get some small change to use. The money I would have used to go to [the hub] is what I use to buy and cook good food to eat. [Also I have] money for offering in church. (Patient Participant, Individual Interview, Female)

*(B) Saving Time*. Participants also placed a high value on the amount of time they saved by attending decentralized clinics. One recalled:

When I remember my [hub] clinic visits, my heart skips. (Patient Participant, Individual Interview, Female)

Like so many patients interviewed for this study, this woman explained she would have to wake up at 4 a.m. in order to get to her clinic visit at the hub. Poor roads, traffic gridlock, and delays at car parks made for long and unpleasant journeys. By transferring to a decentralized clinic, she could now leave home later, walk to the clinic on foot, and return in time for lunch.

Saving time was particularly valuable for individuals whose journeys to the hub required an overnight stay. By transferring to local clinics, patients found their care could be incorporated into the structure of a single normal day.

If I have something doing, I can come as early as possible. If you are the first person on line, you will be treated and you go away to attend to other things. (Patient Participant, Individual Interview, Female)

Another echoed this experience.

Accessing care close home is better. You can leave [and return] home same day. Even if we finish the clinic here at 5 pm, we will be able to go back home. (Patient Participant, Individual Interview, Female)

For many, the time investment required to visit the hub translated into time away from important domestic and paid work activities. Time away had negative economic repercussions and raised suspicion in local communities. It was often difficult for patients to explain long and regular absences to those who depended on their labor, both within and outside the home. Workers resorted to giving excuses or lying to bosses and coworkers to explain away their extended absences. Those formally employed feared having to repeatedly request permission for time away because they felt it put them at risk of losing their jobs. These time constraints and related complications were mitigated by transferring care to decentralized sites.

*(C) “Staying Safe”*. For some participants in this study, the journey to the hub for care was fraught with safety issues associated with the civil unrest centered in the north east (see under Research Setting, above). Roads were regularly blocked by security personnel, making travel difficult and, in some cases, impossible.

‘All the fighting and running all over,' explained one participant, ‘sometimes, there is a crisis and there will be road blocks mounted.' (Patient Participant, Individual Interview, Female)

One participant provided a particularly vivid account of an experience she encountered while travelling home from a hub clinic appointment. She recounted:

…There was a day I came to [the hub] for my medication and after about 12 noon, I headed [home]. Along the way, there was a crisis situation in which vehicles were being stopped along the road. [Members of a local ethnic group] were dragged from the vehicles and killed. I was in a taxi which I [had] entered from the junction and when we got to around the airport…I was the only female in the vehicle. The driver diverted the vehicle and suddenly, I saw the driver drive us to a house with a gate. The other passengers dragged me out; they said it was my type they were looking for. I swore to them I was not [ethnic group]. They didn't believe me. I was tied all round – my hands, legs, neck – and was left in an uncompleted building till about 6 p.m. (Patient Participant, Individual Interview, Female)

This participant was ultimately spared thanks to a sympathetic woman who freed her from the men who, she explained, were “*bent on killing me.*” Her experience dramatically illustrates the risks that patients living in conflict zones undertake in travelling long distances to health care appointments.

*(D) A “Family-Like Atmosphere”*. Decentralized clinics included in this study followed relatively few HIV patients, who lived in nearby communities. The smaller numbers made it easier for people to recognize and get to know one another. In some clinics, they began to build social relationships. The intimate ambiance at certain local clinics resulted in a “family-like atmosphere” that often extended beyond the clinic itself. One interviewee explained:

Elsewhere, apart from the clinic, we greet each other as part of a family. We get to know [each other] because the size here is unlike [the hub] where you meet hundreds of people…The number is manageable, so we can know ourselves…. (Patient Participant, Individual Interview, Female)

Development of relationships among patients was deliberately reinforced by staff, who promoted the formation of support networks through organized group meetings, positive living discussions, and other activities offering the opportunity to socialize in a congenial atmosphere. A provider participant explained that these meetings were spaces where patients could:

…encourage and tell themselves ways someone can live a positive life, encourage [each other] on diet, [and] taking their drugs promptly. [They] help themselves, they share their problems with other people and among themselves. They give themselves advice on what and what not to do. (Provider Participant, Male)

At some sites, providers explicitly counseled patients to respect one another as they would a member of their own family and to protect the privacy of their peers both within and outside of the clinic. A patient participant reported:

…they [providers] talk about keeping each other's secrets. When we meet other people we saw here outside the hospital, we are not to go about revealing their HIV status…. (Patient Participant, Individual Interview, Female)

Some providers at decentralized clinics acted as matchmakers for HIV-positive patients who wished to marry but felt unable to do so due to their HIV status. They attended match-made weddings of these patients as part of the “family.” One provider explained her role in these clinic-based marriages this way:

They [patients] sometimes call me to meet them. Some will come boldly to meet me and say, ‘this is my test, I am HIV positive, I want to marry, how can I get someone to marry?' I collect their phone numbers and [match make] them. They discuss and if they are [compatible], they go ahead to marry. I make people feel free. (Provider Participant, Male)

#### 3.2.2. Perceived Disadvantages of Decentralization

*(E) Loss of Ancillary Services*. Clinic days at the hub began with “health talks”—provider-led educational sessions covering topics related to healthy living with HIV (e.g., ART adherence, nutrition, and hygiene). These sessions were highly valued by patients, who appreciated the opportunity to learn how to live healthier lives. Not every decentralized clinic offered content-rich “health talks.” Where they were not replicated, they were missed, as we see from the following:

Since I left the [hub], I have never gotten a health talk like the ones I did there. What we didn't know we knew [after the talk], so your knowledge increased. If you asked questions, they gave answers. Every time I come to the clinic and I don't listen to a health talk, it is as if I didn't come to the clinic. This is because you will hear what you have never heard before and it will help you. So if you don't hear it on that day, you will not be happy. (Patient Participant, Focus Group, Female)

Also missed were a variety of supplements to HIV care distributed at various times by the hub but not made available to patients at decentralized care sites. Mosquito nets, buckets, infant formula, and cash handouts were all cited by interviewees as valued items they had received for a time as hub patients, but no longer had access to after they changed sites.

Finally, interviewees reported losing access to free medicines for treating non-HIV-related complaints. As hub patients, they discussed whatever complaints they had in clinic visits, often receiving diagnoses and medicines immediately and without charge. At decentralized clinics, drugs other than ART were not available and had to be purchased at outside pharmacies, which presented an economic hardship for many. As one individual explained:

…In [the hub], if you say you are sick, they may just give you the drugs right there in the office. But here [de-centralized clinic], if you are sick they write the drugs for you to go and buy. And if you don't have the money, then you have to go and borrow. Because, if you don't [borrow] your body will suffer. (Patient Participant, Individual Interview, Female)

*(F) Reduced Contact with Health Care Professionals*. Following the health talk, clinic visits at the hub unfolded in a series of steps that brought patients into contact with a variety of health care professionals, including a physician or medical officer. Patients had ample opportunity to discuss their health concerns in group and individual interactions with clinicians and to raise questions related and unrelated to HIV.

With the transfer to decentralized care, these opportunities were often sharply reduced. Decentralized facilities were smaller and had fewer staff, which meant less contact with providers, and a less comprehensive service. One interviewee described the transactional nature of his decentralized care experience this way:

Here, you just enter, collect your drugs, then leave. I don't think they entertain questions. Most of the time [is] on the queue. You enter, collect your drugs, and the next person enters. So I think there is not much time, compared with [the hub]. (Patient Participant, Individual Interview, Male)

*(G) Increased Disclosure Risk*. A third disadvantage of decentralized care cited by patient participants was feeling an increased risk of disclosure of HIV status. This increased risk could be traced to structural aspects of HIV care delivery—both the scheduling of HIV clinic days and the physical layout of clinics themselves.

Because they served relatively small numbers of HIV patients, some decentralized care sites offered HIV clinics only on designated days of the week. This became known in surrounding communities, creating a situation in which individuals seen visiting the clinic on those days could be identified as being HIV-positive. This made it possible for anyone suspecting a family member or friend of having HIV to confirm their suspicions by observing who came and went from the clinic on “HIV days.” One woman described her mother's experience of being “outed” by a relative while keeping a clinic appointment.

… He [relative] asked where people collect drugs and he traced her. When he came to [the waiting area], he saw her sitting down. He followed her to the hospital and inquired about this place. She was worried and confused. Before she knew it, she was drenched in her own sweat [from anxiety]. (Patient Participant, Individual Interview, Female)

The often small size of decentralized care sites can also increase disclosure risk. For example, clinics lacking space for an interior waiting area may have no option but to ask patients to wait outside. Depending on where the outside waiting area is located, patients may find themselves in full view of their acquaintances and neighbors. This was the case at one study site, where the outside waiting area bordered a busy access road. One participant complained to the interviewers:

Can you see this road? People follow it, [and] begin to point fingers at us sitting here. They say, ‘these people have this sickness [HIV].' (Patient Participant, Focus Group, Female)

#### 3.2.3. Expressed Preferences for Hub versus Decentralized Care

As part of the individual interviews, patient participants were asked to state a preference for hub versus decentralized care. Despite the disadvantages described above, a large majority (87%) of those answering this question expressed a preference for decentralized services (see Table 2). Many elaborated on their stated preference. For example, one interviewee said:

…Yes, I used to go to [the hub] and sleep on the floor [the night before the appointment]. Let the government go ahead to open up new places to make access easier…since this HIV is everywhere. The transfer is good and decentralization is good. (Patient Participant, Individual Interview, Female)

## 4. Discussion

This qualitative study examined patient experiences of decentralized HIV care and treatment in the Plateau State Decentralization Initiative, north central Nigeria. The movement to decentralize care and treatment for HIV in Africa has over time evolved into a concern that HIV care be not only decentralized but “differentiated.”

The differentiated HIV care framework prioritizes a “people-centered” approach in which the needs of patients are paramount in the determination and design of service packages. To deliver such acceptable and responsive care it is vital that the needs and priorities of a given population be well understood.

To understand how the delivery of decentralized HIV care is responding to the needs and priorities of patients in north central Nigeria, our research captured a set of advantages and disadvantages that represent patients' key experiences with the decentralization of their own care services. The advantages of decentralization include saving time and money, avoiding threats to safety for individuals living near conflict zones, and experiencing a more intimate “family-like atmosphere” at smaller clinics. Loss of ancillary services, reduced contact with health care professionals, and increased risk of disclosure were identified as disadvantages of decentralized care.

Examined together, patients viewed decentralization as a series of trade-offs. Smaller clinics embedded in local communities could result in a more personalized care experience; these same characteristics raised the risk of unwanted disclosure. Access to care closer to home saved time and money and increased personal security, but at some expense to perceived service quality. Despite these trade-offs, patient participants expressed a strong overall preference for decentralized care.

The time and expense of travelling long distances to keep clinic appointments are widely recognized as a major barrier to accessing HIV care and treatment in sub-Saharan Africa [31–34]. Decentralization addresses this barrier by relocating services to local clinics, closer to where patients live. Despite cost savings, not everyone wishes to receive HIV care locally, as evidenced by low uptake of decentralized service options in some African locations [35]. For our rural participants, however, saving time and money on travel was seen as an important advantage of decentralized HIV care. The cost of transport and travel expenses were a significant burden on these families, and out-of-pocket travel costs were compounded by lost wages or lost labor within the home and the fields. Decentralized care facilitated the recuperation of monetary costs and labor hours, reducing financial worries and allowing individuals to funnel their scarce resources toward the fulfillment of other economic and social responsibilities.

A travel-related access barrier not as widely reported is threats to personal security stemming from the need to move through conflict zones to keep clinic appointments. This kind of travel difficulty figured clearly in the care experiences of some patient participants in this study. Similar experiences are referenced briefly as part of a recent analysis of health service resiliency in northern Nigeria during the Boko Haram insurgency [36]. Our data highlight the very real security risks that individuals face in travelling for care. On a broader scale, the insurgency has resulted in the widespread displacement of civilians in northern Nigeria. This has significantly impacted infectious disease transmission and care, with reports detailing how the insurgency has catalyzed outbreaks such as measles [37] and hampered the delivery of HIV care for the internally displaced [38].

Some decentralized clinics in this study integrated HIV care into their services by offering HIV clinics on particular days of the week. The predictable regularity of clinic visits for HIV in these facilities, as well as the physical layout of certain clinics, left many participants fearing unwanted disclosure of HIV infection. Heightened concerns about disclosure and resulting stigma are also evident in other studies of patient experiences or attitudes toward decentralized care [39, 40]. A recent qualitative study detailing reasons for disengagement from HIV care by Tanzanian women during and after pregnancy shows clearly how clinical facilities that formally or informally separate HIV-related care from other services compromise privacy and increase disclosure risk [41]. More complete integration of HIV treatment and care with other services and improvements to physical plant in decentralized clinics could substantially reduce disclosure risk. These suggestions entail relatively minor investments in infrastructure and a reorganization of care delivery, yet they stand to significantly improve how patients experience decentralized HIV treatment and care.

The few previously published studies addressing patient experiences of provider relationships in decentralized HIV care show mixed results. Some describe disrespectful or otherwise negative attitudes toward patients on the part of decentralized care staff; at least one found better patient-provider relationships in local care sites [39, 40, 42]. Poor treatment at the hands of decentralized clinic staff was not reported by participants in this study. Rather they emphasized the competence and investment in care exhibited by decentralized clinic staff, attributing any less favorable experiences to structural challenges.

The fact that participants cited small patient populations and a “family-like atmosphere” as advantages of decentralized care, while also characterizing reduced opportunities for interactions with health care professionals as a disadvantage, may appear contradictory. However, the “family-like atmosphere” described here stemmed largely from interactions among patients, as these were shaped by individual providers and had little to do with the absolute number or availability of clinical staff.

This research has the following limitations. First, all patient participants were individuals who had chosen to transfer their care to a decentralized site; those who declined transfer are not represented in the sample. Second, this is a qualitative study and therefore the results presented here are not generalizable. We have made efforts to represent study results in broad language that could be meaningful and applicable in other settings, especially small, close-knit communities where decentralized care teams must balance the benefits of being “known” to staff and peers with the heightened risk of unwanted disclosure and stigmatization. Finally, the results may be subject to social response bias in the sense that patients may not have felt completely free to express discontent with decentralized services.

## 5. Conclusion

As HIV service delivery turns increasingly toward community-based, “people-centered” care, the importance of understanding the specific needs and preferences of the communities being served becomes increasingly clear. In studying decentralization from the perspective of “end-users,” we have been able to represent the lived experiences of transitioning from a large, urban “hub” HIV clinic to more local, community-based services for a sample of north central Nigerians living with HIV and a subset of their care providers. These experiences revealed a strong preference for decentralized care, despite important trade-offs, pointing to the central importance for these patients of being able to access services closer to home. Our findings reconfirm difficulty and cost of travel as a fundamental barrier to HIV care for patients living outside urban centers in resource-scarce locations and suggest the transition to community-based care will be enthusiastically received.

## Figures and Tables

**Table 1:** Personal information on study patient participants.

	Study participants (*N* = 93)
	N (%) or median (IQR)
*Sex (N* = 93)	
Male	22 (24%)
Female	71 (76%)
*Marital status (N* = 93)	
Married	64 (69%)
Single	2 (2%)
Widowed	18 (19%)
Separated or divorced	9 (10%)
*Age (years) (N* = 90)	38 (30–45)
*Education (years) (N* = 86)	9 (6–14)

**Table 2:** Preferences for hub versus decentralized care expressed by patient participants in individual interviews.

Variable	N (%)
Preference for decentralized care	52 (87%)
Preference for care at the hub	6 (10%)
No preference	2 (3%)
Missing data	10 (10%)

Total	70
